# Correction to: An Efficient Index for Reachability Queries in Public Transport Networks

**DOI:** 10.1007/978-3-030-54832-2_17

**Published:** 2020-08-17

**Authors:** Bezaye Tesfaye, Nikolaus Augsten, Mateusz Pawlik, Michael H. Böhlen, Christian S. Jensen

**Affiliations:** 8 ERIC (Research Unit 3083), Université Lumière Lyon 2, Lyon, France; 9 National Research University, Higher School of Economics, St. Petersburg, Russia; 10 Poznan University of Technology, Poznań, Poland; 11 University of Salzburg, Salzburg, Austria; 12 University of Zurich, Zürich, Switzerland; 13 Aalborg University, Aalborg, Denmark

## Correction to: Chapter “An Efficient Index for Reachability Queries in Public Transport Networks” in: J. Darmont et al. (Eds.): *Advances in Databases and Information Systems*, LNCS 12245, https://doi.org/10.1007/978-3-030-54832-2_5

The chapter was originally published without open access. With the author(s)’ decision to opt for retrospective open access the copyright of the chapter changed to © The Author(s) 2020 and the chapter is now available under a CC BY 4.0 license at link.springer.com.

